# Co-Application of Tetramycin and Chitosan in Controlling Leaf Spot Disease of Kiwifruit and Enhancing Its Resistance, Photosynthesis, Quality and Amino Acids

**DOI:** 10.3390/biom12040500

**Published:** 2022-03-25

**Authors:** Cheng Zhang, Haitao Li, Xiaomao Wu, Yue Su, Youhua Long

**Affiliations:** 1 Guizhou Food Quality and Safety Technology Service Platform, School of Public Health, Guizhou Medical University, Guiyang 550025, China; chengz76@aliyun.com; 2 Department of Agricultural Engineering, Guizhou Vocational College of Agriculture, Qingzhen 551400, China; lisea02@126.com; 3 Research Center for Engineering Technology of Kiwifruit, Institute of Crop Protection, College of Agriculture, Guizhou University, Guiyang 550025, China; xmwu@gzu.edu.cn

**Keywords:** kiwifruit, tetramycin, chitosan, *Lasiodiplodia theobromae*, *Alternaria tenuissima*, leaf spot disease

## Abstract

Leaf spot disease caused by *Lasiodiplodia theobromae* and *Alternaria tenuissima* is a seriously fungal disease in kiwifruit production. In this study, the co-application of tetramycin and chitosan against leaf spot disease in kiwifruit and its effects on the disease resistance, photosynthesis, quality and amino acids of kiwifruit were investigated. The results show that tetramycin exhibited an excellent antifungal activity against *L. theobromae* and *A. tenuissima* with EC_50_ values of 2.37 and 0.16 mg kg^−1^. In the field, the foliar co-application of tetramycin and chitosan could effectively control leaf spot disease with control efficacy of 89.44% by spraying 0.3% tetramycin aqueous solutions (AS) 5000 time liquid + chitosan 100 time liquid, which was significantly (ANOVA, *p* < 0.01) higher than 79.80% of 0.3% tetramycin AS 5000 time liquid and 56.61% of chitosan 100 time liquid. Simultaneously, the co-application of tetramycin and chitosan was more effective than tetramycin or chitosan alone in enhancing the disease resistance and photosynthesis of kiwifruit leaves, as well as improving the quality and amino acids of kiwifruit fruits. This work highlights that chitosan is a practicable, cost-effective and eco-friendly adjuvant of tetramycin for controlling leaf spot disease of kiwifruit, enhancing resistance and photosynthesis of leaves and improving quality and amino acids of fruits.

## 1. Introduction

Kiwifruit (*Actinidia chinensis*), a typical third-generation fruit rich in vitamin C, essential amino acids for humans and various minerals, has high nutritional, medicinal and economical values [1,2,3]. As a momentous industry for reducing poverty and revitalizing rural areas, the kiwifruit industry has developed rapidly in China, especially in the Guizhou Province of southwest China, where the planting area had reached over 40,000 ha [2,3]. However, leaf spot disease caused by fungal pathogens, such as *Lasiodiplodia theobromae*, *Alternaria tenuissima*, *Corynespora cassiicola*, *Pseudocercospora actinidiae* *cingulat*, *Phomopsis* sp. and *Glomerella cingulate*, occurs frequently and seriously affects the quality and yield of kiwifruit [4,5,6,7,8,9]. Our previous studies found that leaf spot diseases caused by *L. theobromae* and *A. tenuissima* were seriously fungal diseases of kiwifruit production area in the Guizhou Province, whose initial stage was in late June and whose peak occurred from July to August [8,9]. Leaf spot disease seriously affects the photosynthesis of kiwifruit leaves and the growth of kiwifruit plants, thereby resulting in the decline of kiwifruit yield and quality, as well as major economic losses. As a consequence, there is an urgent need to excogitate a practicable, cost-effective and environmentally friendly control strategy against leaf spot disease of kiwifruit.

Currently, chemical fungicides with high efficiency and low toxicity are still the most effective and frequent measures for controlling plant diseases. Nevertheless, it is generally understood that chemical fungicide residuals are potentially harmful to the environment, wild life and human beings. Simultaneously, these chemical fungicides tend to easily generate the resistance of pathogens with increasing application frequency [10,11]. Consequently, developing alternative practices of chemical control has a great practical significance for the sustainable development of the kiwifruit industry. Compared with chemical fungicides, natural products are mild and basically harmless, which has been suggested as one of the reasons for their growing popularity among consumers and farmers, and their increasingly popular use in agriculture [12,13]. Nonetheless, natural products are often less effective than chemical fungicides in controlling plant diseases. In this situation, looking at whether the synergistic application of natural products can more effectively control plant diseases and avoid the resistance of pathogens to chemical fungicides is certainly worth further exploration.

Tetramycin, a novel medical or agricultural antibiotic, is the metabolite of *Streptomyces hygrospinosus* var. beijingensis [14,15]. Tetramycin has been widely used in agricultural plant protection in recent years due to its promising antimicrobial activity and eco-friendly advantage. It has been demonstrated to possess satisfactory bioactivity against various plant-pathogenic fungi, such as *Botrytis cinerea*, *Colletotrichum scovillei*, *Pyricularia oryzae*, *Phytophthora capsici* and *Passalora fulva* [16,17,18,19,20,21]. In our previous study, Wang et al. reported that tetramycin exhibited the superior antibacterial and antifungal activity against various kiwifruit pathogens, including *Pseudomonas syringae* pv. *actinidiae*, *Pseudomonas fulva*, *Agrobacterium tumefaciens*, *Botryosphaeria dothidea*, *Phomopsis* sp., *Alternaria tenuissima*, *Armillariella mellea* and *Phytophthora cactorum* [3]. Additionally, tetramycin application notably increased disease resistance of kiwifruit fruits, as well as effectively enhanced fruit growth, quality and storability. In China, tetramycin is gradually becoming an optimized alternative to chemical fungicides or conventional antibiotics for controlling fruit, vegetable and rice diseases [22,23]. However, the long-term single use of tetramycin may increase environmental antibiotic resistance, leading to its ban or restricted use. 

Chitosan, a natural, antibacterial, nontoxic, antioxidant, renewable and biocompatible compound, has been widely used in agriculture, food, medicine and cosmetic fields [24,25,26]. Many studies have demonstrated that chitosan is a bio-fungicide, resistance inductor and growth enhancer for controlling plant diseases and promoting plant growth [24,25,26,27,28,29]. For instance, Rusin et al. [30] reported that chitosan had a potential toxic activity against *L. theobromae*. Li et al. [31] found that the foliar application of chitosan could effectively control powdery mildew of *Rosa roxburghii* and enhance its photosynthesis and quality. In our previous study, Wang et al. [32] also reported chitosan could enhance tetramycin against soft rot of kiwifruit and promote tetramycin’s improvement for the yield, quality and aroma of kiwifruit fruits. Accordingly, it is worth further studying whether the co-application of tetramycin and chitosan can effectively control leaf spot disease of kiwifruit, enhance the disease resistance and photosynthetic characteristics of kiwifruit leaves, improve amino acids of kiwifruit fruits, as well as avoid the risks of environmental antibiotic resistance.

In this study, the control efficacy of the co-application of tetramycin and chitosan against leaf spot disease of kiwifruit was firstly evaluated. Subsequently, the effects of the co-application of tetramycin and chitosan on the disease resistance, photosynthesis, quality and amino acids of kiwifruit were also investigated. This work provides a practicable, green, safe and efficient approach for controlling leaf spot disease of kiwifruit and enhancing its photosynthesis, quality and amino acids.

## 2. Materials and Methods

### 2.1. Pathogens, Fungicides and Culture Medium

*Lasiodiplodia theobromae* and *Alternaria tenuissima* with high pathogenicity were provided by the Research Center for Engineering Technology of Kiwifruit, Guizhou University, Guizhou Province, China. An amount of 0.3% Tetramycin aqueous solutions (AS) was obtained from Microke Biological Engineering Co. Ltd., Liaoning, China. Chitosan (Deacetylation ≥ 90.00%) was provided from Huarun bioengineering Co. Ltd., Zhenzhou, China. Additionally, potato dextrose agar culture medium (PDA, 200 g potato, 20 g dextrose, 15 g agar and 1000 mL distilled water) was sterilized at 121 °C for 30 min, and its pH value was 6.53.

### 2.2. Toxicity Tests of Tetramycin and Chitosan In Vitro

The toxicities of tetramycin or chitosan against *L. theobromae* and *A. tenuissima* were determined by the mycelium growth rate method [3]. The tested solutions of tetramycin or chitosan at five gradient levels were prepared with sterile water. An amount of 1 mL tested solutions of tetramycin or chitosan were uniformity mixed into 9 mL fresh PDA liquid (45~55 °C), and the control was 1 mL sterile water. Additionally, the mixed fungicide-PDA liquid was then fed in the Petri dishes of 90 mm diameter and left until solidified. Subsequently, a 5 mm pathogen disc was cut from a 7-day-old *L. theobromae* or *A. tenuissima* PDA plate and then placed in the aforementioned solidified PDA center, with the mycelium side down and three replicates. After the treated plates were cultured at 28 °C for 2 days, the diameters of *L. theobromae* or *A. tenuissima* growth in the PDA plates were determined by the criss-crossing method. The growth inhibition of *L. theobromae* or *A. tenuissima* by tetramycin and chitosan was calculated according to Equation (1):

Inhibition rate (%) = 100 × [(Mycelium diameter of control − Mycelium diameter of treatment)/(Mycelium diameter of control − 5)](1)

The linear regression equations and EC_50_ (effective concentration of 50% inhibition rate) values of tetramycin or chitosan against *L. theobromae* and *A. tenuissima* were calculated using the SPSS 18.0 software. Briefly, the five inhibition rates of tetramycin or chitosan against the pathogen were determined by five gradient concentrations, which were set according to the pre-experiment results, respectively. Then, the linear regression equation and EC_50_ value were calculated by five-series concentrations of fungicides and their corresponding five inhibition rates on pathogens.

### 2.3. Field Control Experiment of Leaf Spot Disease

The control experiment of leaf spot disease by tetramycin+chitosan, tetramycin or chitosan was conducted in a kiwifruit orchard with a 5-year-old “Guichang” cultivar in Shidong Town, Xifeng country, Guizhou Province, China (27°04′, 106°55′). The proportion of the female and male plants in kiwifruit orchard was 8:1, and its plant spacing was 3.00 m × 3.00 m. The mean altitude, temperature and annual rainfall of the kiwifruit orchard was about 1250 m, 12.5 °C and 1203 mm, respectively. The physical and chemical characteristics of planting soils (0~60 cm deep) are shown in Table 1.

The foliar spray method was used for the control experiment of leaf spot disease. The four treatments were 0.3% tetramycin AS 5000 time + chitosan 100 time dilution liquid, 0.3% tetramycin AS 5000 time dilution liquid, chitosan 100 time dilution liquid and clear water (control), respectively. A total of twelve plots were arranged randomly with three replicates, and each plot had nine trees and five trees on the diagonal, which were used for measuring. Considering leaf spot disease mainly began to damage kiwifruit leaves in late June every year, reaching a peak in August, accordingly, about 1.00 L of fungicide dilution liquid was sprayed on kiwifruit plants (including leaves, stems and fruits) on 25 June and 25 July 2021, respectively.

The disease index and control effect of leaf spot disease in kiwifruit were investigated on 25 August 2021 according to Zhao et al. [4], with slight modifications. Twenty leaves in the east, west, south, north and middle parts of each tested tree were used for investigation. The disease grade values were: 0 = no incidence, 1 = the diseased spot area accounted for less than 10% of the whole leaf area, 3 = the diseased spot area accounted for 11~25% of the whole leaf area, 5 = the diseased spot area accounted for 26~40% of the whole leaf area, 7 = the diseased spot area accounted for 41~65% of the whole leaf area, 9 = the diseased spot area accounted for more than 65% of the whole leaf area. The disease indices and control effects of tetramycin + chitosan, tetramycin or chitosan for leaf spot disease in kiwifruit were calculated in Equations (2) and (3), respectively.

Disease index = 100 × ∑ (Disease grade value × Leaves of each grade)/(Total number of leaves × the highest grade)(2)

Control effect (%) = 100 × (1 − Disease index of treatment/Disease index of control)(3)

The pathogens of leaf spot disease of kiwifruit in the experimental orchard were isolated by the tissue separation method according to Shi et al. [8]. In the ultra-clean worktable, 3 mm × 3 mm lesion tissues were cut from the junction of disease and health in diseased leaves by a sterile scalpel, soaked in 75% alcohol for disinfection for 30~35 s and then washed with sterile water 3 times and transferred to the sterile filter paper to drain the water. Subsequently, the lesion tissue was transferred to the fresh PDA plate, and the plate was cultured at 28 °C until the colonies developed and were then purified. Two strains were obtained and named as isolated pathogens YB-1 and YB-2, respectively. Moreover, the morphological characteristics of their conidia were observed through a microscope.

### 2.4. Determination of Disease Resistance Parameters and Photosynthetic Characteristics of Kiwifruit Leaves

The phenolics, flavonoids, soluble protein, malonaldehyde (MDA), resistance enzyme activities and photosynthetic characteristics of kiwifruit leaves were determined on 25 August 2021. Total phenolics and total flavonoids were determined using an UV-5800PC spectrophotometer at 280 nm (OD_280_) and 325 nm (OD_325_) with 20 mL 1% (*v*/*v*) HCl-methyl alcohol at 3 °C for 1 h without light extraction [32]. The soluble protein, MDA, catalase (CAT), peroxidase (POD), polyphenoloxidase (PPO) and superoxide dismutase (SOD) activities of kiwifruit leaves were determined as described by Zhang et al. [33]. The chlorophyll content of kiwifruit leaves was measured using an UV-5800PC spectrophotometer at 645 nm (OD_645_) and 663 nm (OD_663_) with an acetone–ethanol (*v*/*v*, 2:1) extraction. A portable LI-6400XT photosynthesis measurement system (LI-COR Inc., Lincoln, NE, USA) was used for monitoring the photosynthetic rate (Pn) and transpiration rate (Tr) of kiwifruit leaves at 8:00–10:00 a.m. on 25 August 2021. Water use efficiency (WUE) of kiwifruit was Pn/Tr.

### 2.5. Determination of Yield, Quality and Amino Acids of Kiwifruit 

Two kiwifruit fruits from the east, west, south, north and middle parts of each tested tree were collected on 25 September 2021. The growth parameters of kiwifruit fruits, including longitudinal diameter, transverse diameter, lateral diameter, fruit shape index, single fruit volume and single fruit weight, were measured [33]. Simultaneously, fruit quality parameters, such as vitamin C, soluble sugar, soluble solid, dry matter, titratable acidity, were determined as described by Wang et al. [30]. Subsequently, when kiwifruit fruits reached an edible state, their 17 hydrolyzed amino acids were determined by a HPLC system (ThermoFisher U3000, Waltham, MA, USA) according to Zhang et al. [34]. Additionally, sweet, flavor, bitter, aromatic, essential, nonessential and total amino acids were calculated based on the contents of the 17 hydrolyzed amino acids. Sweet amino acids = serine + glycine + histidine + threonine + alanine + proline, flavor amino acids = aspartic + glutamate + lysine, bitter amino acids = arginine + valine + methionine + isoleucine + leucine, aromatic amino acids = cystine + tyrosine + phenylalanine. Essential amino acids = threonine + valine + methionine + isoleucine + leucine + phenylalanine + lysine, nonessential amino acids = aspartic + glutamate + cystine + serine + glycine + histidine + arginine + alanine + tyrosine. Amino acids total was equal to the sum of the 17 hydrolyzed amino acids.

### 2.6. Statistical Analyses

The mean ± standard deviation (SD) of three replicate results of each treatment were exhibited. The variance and normality analyses of data were performed on SPSS 18.0 (SPSS Inc., Chicago, IL, USA). A one-way analysis of variance (ANOVA) was determined for the difference significance of disease control efficacy, as well as resistance, photosynthesis, yield, quality and amino acid parameters. Quantile–quantile (Q-Q) plot test was determined for the normality analysis. Charts were drawn with Origin 10.0. The linear regression equation and EC_50_ value were calculated on SPSS 18.0 software. 

## 3. Results

### 3.1. Toxicity of Tetramycin and Chitosan against Pathogens of Leaf Spot Disease

The toxicities of tetramycin and chitosan against *L. theobromae* and *A. tenuissima* are shown in Table 2. The 0.3% tetramycin AS exhibited an outstanding toxicity against *L. theobromae* and *A. tenuissima* with EC_50_ values of 2.37 and 0.16 mg kg^−1^, which was higher by 44.78- and 558.19-fold compared to chitosan, respectively. Although chitosan had a relatively inferior toxicity against *L. theobromae* and *A. tenuissima*, its EC_50_ values still achieved 106.13 and 89.31 mg kg^−1^, respectively. The results demonstrated that tetramycin and chitosan possessed notable potential to control leaf spot disease of kiwifruit caused by *L. theobromae* and *A. tenuissima*.

### 3.2. Field Control Effect of Tetramycin and Chitosan against Leaf Spot Disease of Kiwifruit

The field control effects of tetramycin + chitosan, tetramycin and chitosan against leaf spot disease of kiwifruit are depicted in Table 3. Tetramycin + chitosan, tetramycin or chitosan significantly (ANOVA, *p* < 0.01) decreased the disease index of leaf spot disease of kiwifruit, among which tetramycin + chitosan was the most effective. The control effect of 0.3% tetramycin AS 5000 time + chitosan 100 time dilution liquid against leaf spot disease was 89.44%, which was significantly (ANOVA, *p* < 0.01) higher than 79.80% of 0.3% tetramycin AS 5000 time liquid and 56.61% of chitosan 100 time liquid. These results demonstrate that chitosan had a preferable induced control effect on leaf spot disease, and its mixed application with tetramycin could significantly improve tetramycin’s control effect on leaf spot disease of kiwifruit.

**Figure 1:**
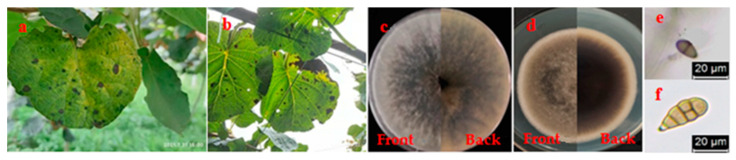
Field symptoms of leaf spot disease of kiwifruit and its pathogens’ morphological characteristics. (**a**): Front side symptoms of leaf spot disease, (**b**): Back side symptoms of leaf spot disease, (**c**): The front and back sides of isolated pathogen YB-1 grown on PDA plate, (**d**): The front and back sides of isolated pathogen YB-2 grown on PDA plate, (**e**): Conidium of isolated pathogen YB-1, (**f**): Conidium of isolated pathogen YB-2.

Field symptoms of leaf spot disease of kiwifruit in the experimental orchard and its pathogens’ morphological characteristics are depicted in Figure 1. The field symptoms (Figure 1a,b) of leaf spot disease of kiwifruit in the experimental orchard were the same as those reported in our previous study [8,9]. Mycelial and conidia morphologies (Figure 1c,e) of isolated pathogen YB-1 were similar to those of *L. theobromae* [8]; meanwhile, mycelial and conidia morphologies (Figure 1d,f) of isolated pathogen YB-2 resembled those of *A. tenuissima* [9]. The results indicated that both *L. theobromae* and *A. tenuissima* were colonized symptomatic kiwifruit leaves.

### 3.3. Effects of Tetramycin and Chitosan on Resistance Compounds and Enzyme Activities of Kiwifruit Leaves

**Figure 2:**
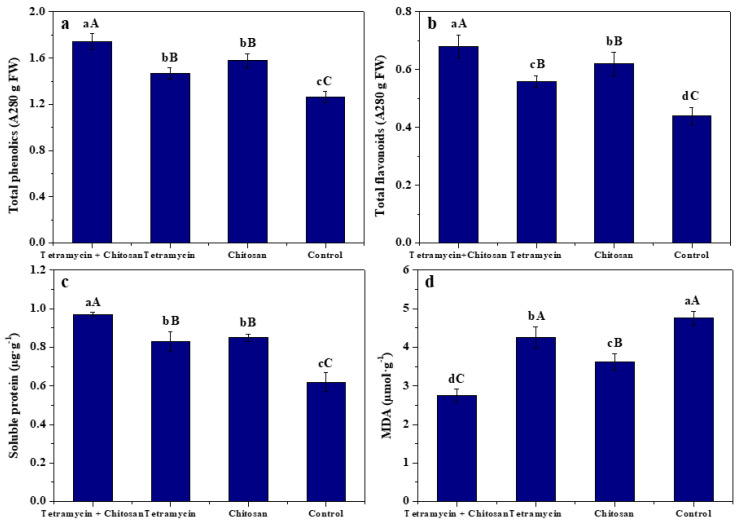
The effects of tetramycin + chitosan, tetramycin and chitosan on the total phenolics (**a**), total flavonoids (**b**), soluble protein (**c**) and MDA (**d)** of kiwifruit leaves. Values and error bars indicate the mean and SD of three replicates, respectively. Different small and capital letters indicate significant differences at 5% level (ANOVA, *p* < 0.05) and 1% level (ANOVA, *p* < 0.01), respectively.

**Figure 3:**
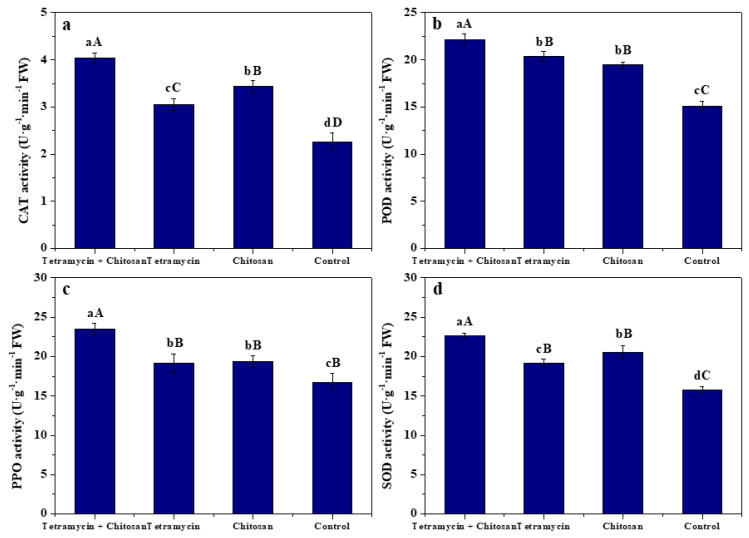
The effects of tetramycin + chitosan, tetramycin and chitosan on the CAT (**a**), POD (**b**), PPO (**c**) and SOD (**d**) activities of kiwifruit leaves. Values and error bars indicate the mean and SD of three replicates, respectively. Different small and capital letters indicate significant differences at 5% level (ANOVA, *p* < 0.05) and 1% level (ANOVA, *p* < 0.01), respectively.

The effects of tetramycin + chitosan, tetramycin and chitosan on the total phenolics, total flavonoids, soluble protein, MDA, CAT, POD, PPO and SOD activities of kiwifruit leaves are displayed in Figure 2 and Figure 3. Compared with tetramycin, chitosan and control, tetramycin + chitosan significantly (ANOVA, *p* < 0.01) enhanced total phenolics, total flavonoids, soluble protein, CAT, POD, PPO and SOD activities of kiwifruit leaves and effectively decreased leaf MDA content. Simultaneously, compared with control, tetramycin and chitosan also significantly (ANOVA, *p* < 0.01) increased total phenolics, total flavonoids, soluble protein, CAT, POD and SOD activities of kiwifruit leaves and significantly (ANOVA, *p* < 0.05) enhanced leaf PPO activities, as well as significantly (ANOVA, *p* < 0.01) decreasing leaf MDA content. Moreover, compared with tetramycin, chitosan was more likely to significantly (ANOVA, *p* < 0.01) improve total flavonoids and CAT activity of kiwifruit leaves and significantly (ANOVA, *p* < 0.05) enhance leaf SOD activity, as well as significantly (ANOVA, *p* < 0.01) decreasing leaf MDA content. These results demonstrate that the co-application of tetramycin and chitosan could effectively enhance the improvement or inhibition effects of tetramycin or chitosan on the total phenolics, total flavonoids, soluble protein, MDA, resistance enzyme activities of kiwifruit leaves, thereby further enhancing the resistance of kiwifruit against leaf spot disease.

### 3.4. Effects of Tetramycin and Chitosan on Photosynthetic Characteristics of Kiwifruit Leaves 

**Figure 4:**
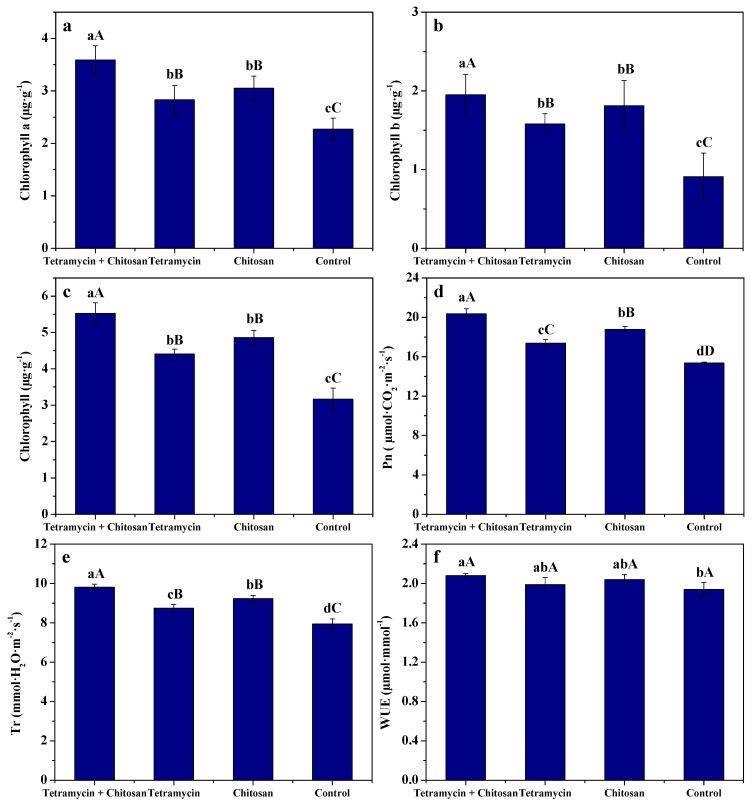
The effects of tetramycin + chitosan, tetramycin and chitosan on the chlorophyll a (**a**), chlorophyll b (**b**), chlorophyll (**c**), photosynthetic rate (**d**), transpiration rate (**e**) and water use efficiency (**f**) of kiwifruit leaves. Values and error bars indicate the mean and SD of three replicates, respectively. Different small and capital letters indicate significant differences at 5% level (ANOVA, *p* < 0.05) and 1% level (ANOVA, *p* < 0.01), respectively.

The effects of tetramycin + chitosan, tetramycin and chitosan on chlorophyll a, chlorophyll b, chlorophyll, photosynthetic rate, transpiration rate and water use efficiency in kiwifruit leaves are displayed in Figure 4. Tetramycin + chitosan, tetramycin and chitosan could effectively improve the chlorophyll a, chlorophyll b, chlorophyll, photosynthetic rate, transpiration rate and water use efficiency of kiwifruit leaves compared with control. The chlorophyll a, chlorophyll b, chlorophyll, photosynthetic rate and transpiration rate of kiwifruit leaves treated by tetramycin + chitosan were significantly (ANOVA, *p* < 0.01) higher or faster than those of kiwifruit leaves treated by chitosan or tetramycin. The photosynthetic rate of kiwifruit leaves treated by chitosan was significantly (ANOVA, *p* < 0.01) faster than that of kiwifruit leaves treated by tetramycin, and its transpiration rate was significantly (ANOVA, *p* < 0.05) faster than that of tetramycin. These results demonstrate that the co-application of tetramycin and chitosan effectively promoted the chlorophyll, photosynthetic rate and transpiration rate of kiwifruit leaves, thereby enhancing their favorable growth.

### 3.5. Effects of Tetramycin and Chitosan on Growth, Quality and Amino Acids of Kiwifruit Fruits

The effects of tetramycin + chitosan, tetramycin and chitosan on kiwifruit fruit growth are shown in Table 4. Tetramycin + chitosan significantly (ANOVA, *p* < 0.05) enhanced the longitudinal diameter, lateral diameter, volume and weight of kiwifruit fruits, which were effectively increased by 0.62, 1.08 or 1.77%, 1.36, 1.50 or 3.37%, 2.45, 3.92 or 6.99% and 2.95, 2.86 or 6.96% compared with tetramycin, chitosan or control treatments, respectively. The transverse diameter and fruit shape index of fruits exhibited no significant (ANOVA, *p* < 0.05) differences in the four treatments. Simultaneously, all growth parameters exhibited no significant (ANOVA, *p* < 0.05) differences in tetramycin and chitosan treatments. These findings demonstrate that the promoted effects of fruit growth by tetramycin + chitosan were better than those by tetramycin or chitosan alone.

The effects of tetramycin + chitosan, tetramycin and chitosan on the nutritional quality of kiwifruit fruits are depicted in Table 5. Tetramycin + chitosan, tetramycin and chitosan could effectively enhance vitamin C, soluble sugar, soluble solid and dry matter of kiwifruit fruits and decrease their titratable acidity compared with control. Soluble sugar, soluble solid and dry matter of kiwifruit fruits treated by tetramycin + chitosan were significantly (ANOVA, *p* < 0.05) higher than those treated by tetramycin, and soluble solid and dry matter of kiwifruit fruits treated by chitosan were significantly (ANOVA, *p* < 0.05) higher than those treated by tetramycin. Simultaneously, all quality parameters exhibited no significant (ANOVA, *p* < 0.05) differences in tetramycin + chitosan and chitosan treatments. These findings demonstrate that chitosan could effectively enhance tetramycin’s improvement effects on the nutritional quality of kiwifruit fruits.

The effects of tetramycin + chitosan, tetramycin and chitosan on amino acids of kiwifruit fruits are displayed in Table 6. Tetramycin + chitosan, tetramycin and chitosan could effectively increase total amino acids of fruits compared with control. Flavor, bitter, essential, nonessential and total amino acids of kiwifruit fruits treated by tetramycin + chitosan were significantly (ANOVA, *p* < 0.05) higher than those treated by tetramycin or control treatments, and aromatic amino acids of tetramycin + chitosan were also significantly (ANOVA, *p* < 0.05) higher than those of tetramycin. Flavor, aromatic, nonessential and total amino acids of kiwifruit fruits treated by chitosan were also significantly (ANOVA, *p* < 0.05) higher than those treated by tetramycin or control treatments, and their essential amino acids were significantly (ANOVA, *p* < 0.05) higher than those of control. These findings demonstrate that the improvement effect of fruit amino acids by tetramycin + chitosan was superior to that of tetramycin or chitosan alone.

## 4. Discussion

Previous findings have demonstrated that tetramycin could effectively inhibit the mycelial growth of many plant pathogens [3,16,17,18,19,20,21], and chitosan also had antifungal activity against various fungal pathogens [24,25,26,27,28,29,30,31,32]. Rusin et al. [30] reported that the mycelial growth of *L. theobromae* was decreased by tebuconazole, mancozeb, pyraclostrobin, fosetyl-Al, *Bacillus Subtilis*, and *Trichoderma harzianum*, which were the most effective, followed by difeconazole and *Trichoderma harzianum*, and sulfur, azoxystrobin, and chitosan did not differ from the control. The results here show that 0.3% tetramycin AS exhibited an excellent toxic activity against *L. theobromae* and *A. tenuissima* with EC_50_ values of 2.37 and 0.16 mg kg^−1^, while chitosan had a relatively inferior toxic activity against those with EC_50_ values of 106.13 and 89.31 mg kg^−1^, respectively. The results in this work extended the antimicrobial spectrum of tetramycin and chitosan. Additionally, the control effect of the foliar co-application of 0.3% tetramycin AS 5000 time + chitosan 100 time dilution liquid against leaf spot disease was 89.44%, which was significantly (ANOVA, *p* < 0.01) higher than 79.80% of 0.3% tetramycin AS 5000 time liquid and 56.61% of chitosan 100 time liquid. Leaf spot disease of kiwifruit occurred seriously in this experimental orchard in recent years. Meanwhile, *L. theobromae* and *A. tenuissima* were isolated from diseased kiwifruit leaves caused by leaf spot disease in the experimental orchard, indicating that the control of leaf spot disease of kiwifruit by the above treatments in the field were effective. In our previous works, Wang et al. [3,32] reported that 0.3% tetramycin AS possessed a good field control efficacy for brown spot disease caused by *A. tenuissima* in kiwifruit, and chitosan could effectively enhance tetramycin against soft rot of kiwifruit. Simultaneously, chitosan can activate plant defense responses by inducing various defense-related reactions [24,25,26,35]. In this study, the co-application of tetramycin and chitosan significantly (ANOVA, *p* < 0.01) decreased the occurrence of leaf spot disease in kiwifruit compared with tetramycin or chitosan alone. This demonstrates that tetramycin and chitosan might have a notable synergetic effect for controlling leaf spot disease of kiwifruit, and this effect was probably derived from the excellent antimicrobial activity of tetramycin, as well as the preferable antimicrobial and induced resistance activity of chitosan.

Phenolics, flavonoids, soluble protein and MDA, as well as CAT, POD, PPO and SOD activities, are closely related to plant disease resistance [36,37,38,39]. Many reports have also demonstrated that chitosan could induce total phenolics, total flavonoids and protein increase, MDA decrease and boost defense enzyme activity, thereby promoting the plant’s disease resistance [24,25,26,27,28,29,30,31,32,33,34,35,36,37,38,39,40]. The present results show that compared with tetramycin, chitosan and control, tetramycin + chitosan significantly (ANOVA, *p* < 0.01) enhanced total phenolics, total flavonoids, soluble protein, CAT, POD, PPO and SOD activities of kiwifruit leaves and effectively decreased leaf MDA content. Our previous results also indicated that chitosan could significantly (ANOVA, *p* < 0.05) improve the promoting effect of tetramycin on SOD activity of kiwifruit fruits and enhance the disease resistance of kiwifruit fruits [31]. These results emphasize that the co-application of tetramycin and chitosan could also effectively enhance the improvement or inhibition effects of tetramycin or chitosan on the total phenolics, total flavonoids, soluble protein, MDA, CAT, POD, PPO and SOD activities of kiwifruit leaves, and it could be more beneficial for improving the disease resistance of kiwifruit leaves.

Chlorophyll is an essential pigment of photosynthesis, and photosynthesis is the physiological basis of plant growth and development, while transpiration is the main driving force for plants to absorb and transport water and nutrients. Chitosan can enhance plant growth and development by promoting the photosynthetic rate by augmenting chlorophyll content [25,26]. The results in this work show that the co-application of tetramycin and chitosan effectively improved the chlorophyll a, chlorophyll b, chlorophyll, photosynthetic rate and transpiration rate of kiwifruit leaves compared with tetramycin, chitosan or control. This favorable effect should be generated by the synergistic effect between tetramycin in protecting the plant’s leaf organs from pathogens and chitosan in promoting the plant’s growth. Dzung et al. [41] reported that chitosan can promote plant growth by triggering the cytokinin and auxin of signal transduction and gene expression, as well as increasing nutrient intake. Our previous study also showed that the foliar application of tetramycin + chitosan or chitosan notably improved growth, quality and aroma of kiwifruit fruits [32]. The results herein demonstrate that the promoting effect of growth and quality of kiwifruit fruits by tetramycin + chitosan was better than that of tetramycin or chitosan alone, which is consistent with the results of our previous studies.

The closer the amino acid composition of foods is to that of the human protein, the higher their nutritional value. According to the amino acid model of the protein nutritional value proposed by WHO and FAO, it is considered that the essential amino acids with superior quality account for about 40% of total amino acids, and the ratio of essential amino acids to nonessential amino acids is more than 0.6 [42]. In this study, the percentage of essential amino acids in total amino acids and the ratio of essential amino acids to nonessential amino acids in kiwifruit fruits treated by tetramycin + chitosan, tetramycin, chitosan and control were 31.54% and 0.53, 30.83% and 0.51, 30.89% and 0.51, and 30.81% and 0.51, respectively. These results demonstrate that the protein nutritional value of kiwifruit fruits treated by tetramycin + chitosan was closer to the ideal mode value than other treatments. Simultaneously, tetramycin + chitosan effectively improved the contents of the sweet, flavor, bitter, aromatic, essential, nonessential and total amino acids in kiwifruit fruits, and their enhancing effect on fruit amino acids was superior to that of tetramycin or chitosan alone. These results highlight that the co-application of tetramycin and chitosan not only improved growth, quality and aroma of kiwifruit fruits, but it also enhanced their amino acids.

At present, increasing attention has been focused and preferred on natural products with high efficacy, nontoxicity and low risk as alternatives to chemical fungicides for controlling plant diseases [43]. Tetramycin is a low-toxicity and eco-friendly natural product, and chitosan is a natural nontoxic substance widely used in food, medicine, cosmetics and other fields [31,32]. Simultaneously, the field application concentration of 0.3% tetramycin AS (5000 time dilution liquid) was very low, and the safe interval (25 July to 25 September, 62 days) and soft ripening (more than 20 days) periods of kiwifruit fruits were also very long. Accordingly, the potential food safety risk caused by tetramycin + chitosan is minute or almost nonexistent. Moreover, to better demonstrate the feasibility of this method, the tetramycin residue analyses of fruits and the safety evaluation and environmental antibiotic resistance of chitosan + tetramycin are our future research topics. This work highlights that the co-application of tetramycin and chitosan can be used as a green, safe, efficient and practicable approach for controlling leaf spot disease of kiwifruit, enhancing its resistance, photosynthesis, quality and amino acids.

## 5. Conclusions

In conclusion, the co-application of tetramycin and chitosan effectively controlled leaf spot disease of kiwifruit and notably promoted total phenolics, total flavonoids, soluble protein, CAT, POD, PPO and SOD activities of kiwifruit leaves and reduced their MDA content, as well as reliably enhancing kiwifruit photosynthesis. Simultaneously, the co-application of tetramycin and chitosan was more effective than tetramycin or chitosan alone in improving the growth, quality and amino acids of kiwifruit fruits. This work highlights that the co-application of tetramycin and chitosan can be used as a feasible practice for controlling leaf spot disease of kiwifruit.

## Figures and Tables

**Table 1:** The physical and chemical characteristics of planting soils of kiwifruit.

Parameters	Content	Parameters	Content
Organic matter	42.36 g kg^−1^	Exchangeable calcium	18.21 cmol kg^−1^
Total nitrogen	1.27 g kg^−1^	Exchangeable magnesium	251.34 mg kg^−1^
Total phosphorus	1.68 g kg^−1^	Available zinc	0.78 mg kg^−1^
Total potassium	1.15 g kg^−1^	Available iron	6.54 mg kg^−1^
Available nitrogen	95.85 mg kg^−1^	Available manganese	14.38 mg kg^−1^
Available phosphorus	16.25 mg kg^−1^	Available boron	0.16 mg kg^−1^
Available potassium	1.46 mg kg^−1^	pH	6.47

**Table 2:** Toxicities of tetramycin and chitosan against *L. theobromae* and *A. tenuissima*.

Pathogens	Fungicides	Regression Equation	Determination Coefficient (*R*^2^)	EC_50_ (mg kg^−1^)
*L. theobromae*	0.3% Tetramycin AS	*y* = 4.5323 + 1.2463*x*	0.9840	2.37
	Chitosan	*y* = 6.0308 + 1.0580*x*	0.9809	106.13
*A. tenuissima*	0.3% Tetramycin AS	*y* = 5.7631 + 0.9705*x*	0.9928	0.16
	Chitosan	*y* = 6.0232 + 0.9754*x*	0.9775	89.31

*y* and *x* indicate the inhibition rate and fungicide concentration, respectively.

**Table 3:** The control effect of tetramycin + chitosan, tetramycin and chitosan against leaf spot disease of kiwifruit.

Treatments	Disease Index	Control Effect (%)
Tetramycin + Chitosan	1.05 ± 0.09 ^dC^	89.44 ± 1.67 ^aA^
Tetramycin	2.01 ± 0.15 ^cC^	79.80 ± 3.06 ^bB^
Chitosan	4.33 ± 0.11 ^bB^	56.61 ± 4.18 ^cC^
Control	10.03 ± 0.78 ^aA^	

The values indicate the mean ± SD of three replicates. Different small and capital letters in the same column indicate significant differences at 5% level (ANOVA, *p* < 0.05) and 1% level (ANOVA, *p* < 0.01), respectively.

**Table 4:** The effects of tetramycin + chitosan, tetramycin and chitosan on the growth of kiwifruit fruits.

Treatments	Longitudinal Diameter (mm)	Transverse Diameter (mm)	Lateral Diameter (mm)	Fruit Shape Index	Single Fruit Volume (cm^3^)	Single Fruit Weight (g)
Tetramycin + Chitosan Tetramycin	76.96 ± 0.50 ^a^	52.84 ± 0.78 ^a^	42.68 ± 0.26 ^a^	1.61 ± 0.03 ^a^	72.68 ± 1.78 ^a^	92.75 ± 0.11 ^a^
	76.48 ± 0.21 ^ab^	52.60 ± 0.44 ^a^	42.10 ± 0.81 ^ab^	1.62 ± 0.01 ^a^	70.90 ± 1.02 ^a^	90.01 ± 0.71 ^b^
Chitosan	76.13 ± 0.15 ^bc^	52.12 ± 0.48 ^a^	42.04 ± 0.27 ^ab^	1.62 ± 0.02 ^a^	69.83 ± 0.94 ^ab^	90.10 ± 0.72 ^b^
Control	75.60 ± 0.24 ^c^	51.78 ± 0.41 ^a^	41.24 ± 0.39 ^b^	1.62 ± 0.02 ^a^	67.60 ± 1.19 ^b^	86.29 ± 1.05 ^c^

Values indicate the mean ± SD of three replicates. Different small letters in the same column indicate significant differences at 5% level (ANOVA, *p* < 0.05).

**Table 5:** The effects of tetramycin + chitosan, tetramycin and chitosan on quality of kiwifruit fruits.

Treatments	Vitamin C (g kg^−1^)	Total Soluble Sugar (%)	Soluble Solid (%)	Dry Matter (%)	Titratable Acidity (%)
Tetramycin + Chitosan Tetramycin	1.92 ± 0.02 ^a^	12.70 ± 0.15 ^a^	15.63 ± 0.06 ^a^	19.65 ± 0.07 ^a^	1.03 ± 0.03 ^b^
	1.88 ± 0.01 ^a^	12.41 ± 0.08 ^b^	15.30 ± 0.10 ^b^	19.18 ± 0.03 ^b^	1.10 ± 0.04 ^a^
Chitosan	1.89 ± 0.01 ^a^	12.63 ± 0.09 ^a^	15.47 ± 0.15 ^ab^	19.50 ± 0.06 ^a^	1.04 ± 0.01 ^b^
Control	1.84 ± 0.01 ^b^	12.07 ± 0.01 ^c^	14.43 ± 0.15 ^c^	18.41 ± 0.13 ^c^	1.16 ± 0.04 ^a^

Values indicate the mean ± SD of three replicates. Different small letters in the same column indicate significant differences at 5% level (ANOVA, *p* < 0.05).

**Table 6:** The effects of tetramycin + chitosan, tetramycin and chitosan on amino acids of kiwifruit fruits.

Amino Acids (g kg^−1^)	Tetramycin + Chitosan	Tetramycin	Chitosan	Control
Aspartic	0.88	0.83	0.85	0.82
Glutamate	1.85	1.83	1.83	1.78
Cystine	0.97	0.94	0.94	0.96
Serine	0.77	0.77	0.76	0.75
Glycine	0.76	0.66	0.74	0.73
Histidine	0.68	0.67	0.67	0.64
Arginine	1.43	1.37	1.40	1.34
Threonine	0.45	0.46	0.47	0.47
Alanine	0.75	0.67	0.72	0.67
Proline	1.24	1.28	1.25	1.29
Tyrosine	0.65	0.65	0.66	0.64
Valine	0.66	0.60	0.64	0.62
Methionine	0.57	0.59	0.55	0.55
Isoleucine	0.61	0.60	0.57	0.55
Leucine	0.63	0.54	0.56	0.56
Phenylalanine	0.75	0.69	0.72	0.68
Lysine	0.94	0.84	0.88	0.86
Sweet amino acids	4.65 ± 0.04 ^a^	4.52 ± 0.09 ^ab^	4.62 ± 0.07 ^ab^	4.55 ± 0.03 ^b^
Flavor amino acids	3.66 ± 0.02 ^a^	3.50 ± 0.03 ^c^	3.56 ± 0.01 ^b^	3.47 ± 0.02 ^c^
Bitter amino acids	3.91 ± 0.05 ^a^	3.70 ± 0.06 ^c^	3.73 ± 0.02 ^b^	3.62 ± 0.01 ^c^
Aromatic amino acids	2.37 ± 0.05 ^a^	2.28 ± 0.06 ^b^	2.33 ± 0.05 ^ab^	2.28 ± 0.01 ^b^
Essential amino acids	4.60 ± 0.02 ^a^	4.32 ± 0.07 ^bc^	4.40 ± 0.04 ^b^	4.29 ± 0.06 ^c^
Nonessential amino acids	8.75 ± 0.08 ^a^	8.40 ± 0.12 ^c^	8.59 ± 0.06 ^b^	8.34 ± 0.04 ^c^
Total amino acids	14.59 ± 0.05 ^a^	14.00 ± 0.07 ^c^	14.23 ± 0.13 ^b^	13.91 ± 0.02 ^c^

Values indicate the mean ± SD of three replicates. Different small letters in the same line indicate significant differences at 5% level (ANOVA, *p* < 0.05).

## Data Availability

The datasets analyzed in the current study are available from the corresponding author on reasonable request.
